# Are Fc Gamma Receptor Polymorphisms Important in HIV-1 Infection Outcomes and Latent Reservoir Size?

**DOI:** 10.3389/fimmu.2021.656894

**Published:** 2021-05-04

**Authors:** Helena Lamptey, Evelyn Y. Bonney, Bright Adu, George B. Kyei

**Affiliations:** ^1^ Department of Immunology, Noguchi Memorial Institute for Medical Research, University of Ghana, Accra, Ghana; ^2^ Department of Virology, Noguchi Memorial Institute for Medical Research, University of Ghana, Accra, Ghana; ^3^ Department of Medicine, Washington University School of Medicine in St Louis, St. Louis, MO, United States; ^4^ Medical and Scientific Research Centre, University of Ghana Medical Centre, University of Ghana, Accra, Ghana

**Keywords:** FcγR polymorphisms, HIV-1 latent reservoirs, HIV-1 cure strategies, HIV-1 disease progression, broadly neutralizing antibodies

## Abstract

Fc gamma receptors (FcγR) are cell surface glycoproteins which trigger specific effector-cell responses when cross-linked with the Fc portions of immunoglobulin (IgG) antibodies. During HIV-1 infection, the course of disease progression, ART response, and viral reservoir size vary in different individuals. Several factors may account for these differences; however, Fc gamma receptor gene polymorphisms, which influence receptor binding to IgG antibodies, are likely to play a key role. FcγRIIa (CD32) was recently reported as a potential marker for latent HIV reservoir, however, this assertion is still inconclusive. Whether FcγR polymorphisms influence the size of the viral reservoir, remains an important question in HIV cure studies. In addition, potential cure or viral suppression methods such as broadly neutralizing antibody (bNAbs) may depend on FcγRs to control the virus. Here, we discuss the current evidence on the potential role played by FcγR polymorphisms in HIV-1 infection, treatment and vaccine trial outcomes. Importantly, we highlight contrasting findings that may be due to multiple factors and the relatively limited data from African populations. We recommend further studies especially in sub-Saharan Africa to confirm the role of FcγRIIa in the establishment of latent reservoir and to determine their influence in therapies involving bNAbs.

## Introduction

An estimated 37.9 million people are infected with the human immunodeficiency virus (HIV). The burden of disease is highest in sub-Saharan Africa, where approximately 25.6 million people live with the virus (1).

For most patients infected with HIV, antibodies elicited by the host immune system have limited potential to neutralize the virus. The result is a gradual decline of host CD4+ T cells leading to full blown acquired immune deficiency syndrome (AIDS) over time. Antiretroviral therapy (ART) is able to suppress the virus and reverse the decline in CD4 count in most patients. However, ART does not provide cure due to a stable latent reservoir established early in the infection process in resting CD4+ T cells, macrophages and other cells.

The course of HIV-1 infection, disease progression, ART response, and reservoir size vary in different individuals. For instance, long term non-progressors can maintain high CD4 count, and control the virus for up to 10 years without ART (2–4), compared to “rapid progressors” who develop full blown AIDS within 3-4 years of infection (5, 6). Then, there is the rare group of HIV-1 infected individuals called “elite controllers”, who can maintain a viral load of less than 50 copies/ml without ART for even longer periods (7, 8). These elite controllers and long term non-progressors, have a smaller viral reservoir (9, 10). Viral factors such as deletions or mutations in key viral genes have been implicated in the differences in natural HIV control. Factors such as source of HIV infection, timing of ART and ethnicity have all been cited as potential determinants of viral reservoir size (11). However, host genetic factors such as human leukocyte antigen (HLA) and polymorphisms in Fc gamma receptor (*FCGR*) genes which influence the receptor binding to immunoglobulin (Ig) G antibodies are likely to be even more critical. FcγR gene copy number variations (CNVs) and/or single nucleotide polymorphisms (SNPs) could cause differences in Fc gamma receptor (FcγR) expression density on effector cell surface, binding affinity to IgG subclasses and signaling potential which would influence HIV-1 infection risk, disease progression and vaccine efficacy (12, 13). In this review, we summarize current knowledge on the role of FcγR gene polymorphisms and HIV-1 infection, in relation to ART outcomes and control of the viral reservoir. We will explore the idea that FcγR polymorphisms could help explain the differences in HIV-1 infection outcomes, responses to ART and broadly neutralizing antibodies (bNAbs) and influence the size of the viral reservoir.

## Overview of FcγRs and Their Role in Host Immunity

FcγRs are cell surface glycoproteins that bind the Fc portions of different IgG subclasses to trigger different cell effector functions (14, 15). FcγRs are expressed on most immune cells including monocytes, natural killer (NK) cells, B cells, eosinophils, basophils, dendritic cells, platelets, macrophages, and some subpopulations of T cells (16–18).

**Figure 1:**
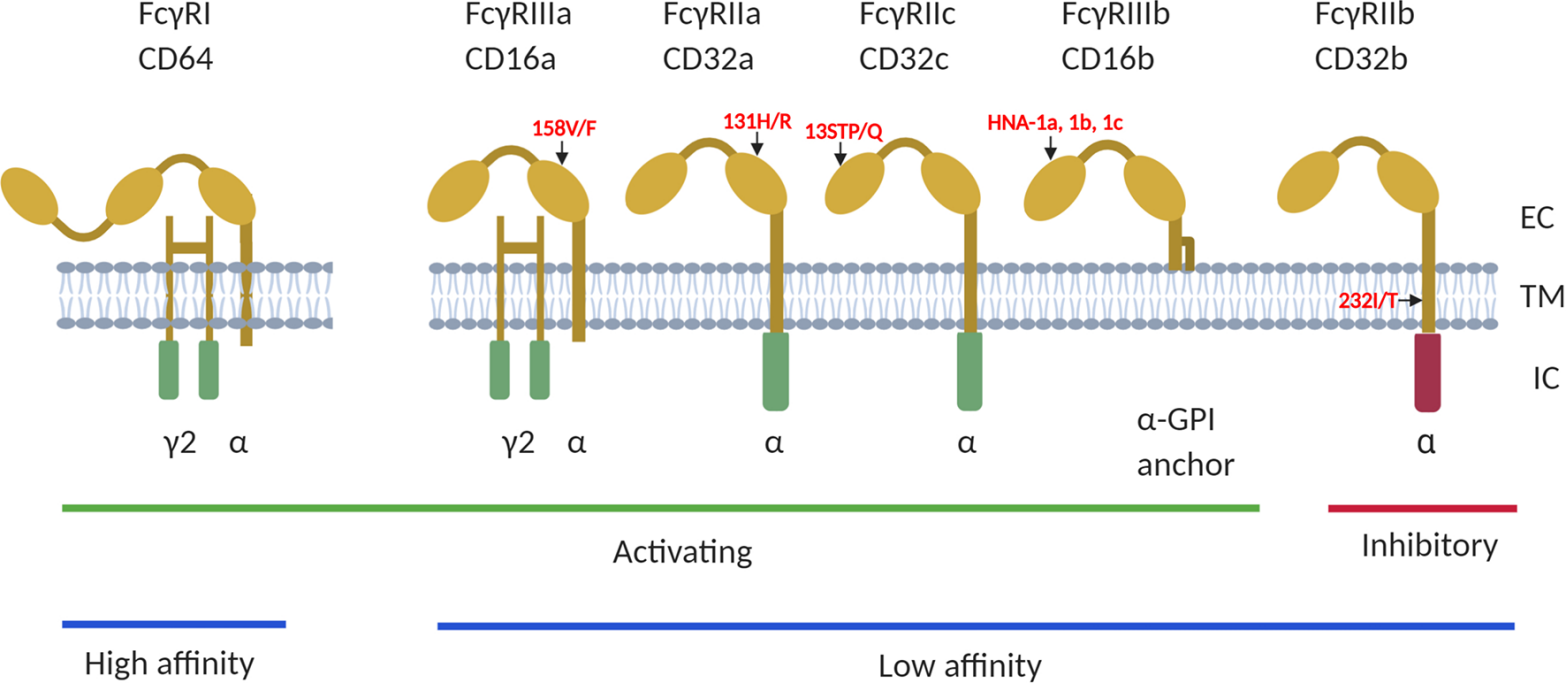
The human FCGRs consists of 3 classes; FcγRI (CD64), FcγRII (CD32) and FcγRIII (CD16), of which FcγRI has high affinity to bind to antibody Fc-fragment. FcγRII and FcγRIII have low affinity for IgG binding. They are also classified as activating or inhibitory due to the signals induced by FcR crosslinking. The FcγRI, FcγRIIa/IIc and FcγRIIIa and FcγRIIIb proteins contain the immunoreceptor tyrosine based activating motifs (ITAM), whiles FcγRIIb is the only receptor that contains the immunoreceptor tyrosine based inhibitory motif (ITIM), in its cytoplasmic domain. Additionally, the FcγRIIIb gene encodes a glycosylphosphatidylinositol (GPI) -anchored receptor, and this encodes human neutrophil specific antigen 1 (HNA1a, b and c). Several functional polymorphisms exist in the FcγR genes, including the FcγRIIa-131H/R of which the 131-H allele has a high binding affinity to IgG2 and FcγRIIIa-176V/F with the 176-V allele being more effective at mediating phagocytosis. The FcγRIIc gene encodes either a glutamine (Q) or a stop codon (STP) at position 13 while the 232-T allele of the FcγRIIb 232I/T polymorphism has a reduced inhibitory signaling capacity. EC, extracellular domain; TM, transmembrane domain; IC, intracellular domain. Summaries of studies that have investigated different interactions between the receptors on different HIV infection outcomes have been provided in **Tables 1**, **2**.

There are three main classes of FcγRs namely FcγRI (CD64), FcγRII (CD32) and FcγRIII (CD16) each with different isoforms encoded by different genes (**Figure 1**). The FcγRI family of receptors consist of 3 genes (*FCGR1A*, *FCGR1B* and *FCGR1C*) that share about 98% sequence homology and thought to flank the centromere of chromosome 1 at bands 1p12 (*FCGR1B*) and 1q21 (*FCGR1A* and *FCGR1C*) (19). FcγRIA is the only known high affinity FcγR. It is expressed by monocytes, dendritic cells (DC’s), macrophages and neutrophils (20) and plays a role in antibody mediated phagocytosis. The FcγRII family of receptors have low binding affinity for IgG and are encoded by three genes (*FCGR2A, FCGR2B* and *FCGR2C*) located on chromosome 1q23.3 (16, 21). They are expressed on neutrophils, DC’s, monocytes, B cells, NK cells, myeloid cells, and platelets. FcγRII family of receptors do not use the common Fcγ-chain for activation (FcγRIIa and FcγRIIc) or inhibitory (FcγRIIb) signaling because their Immunoreceptor Tyrosine-based Activation Motifs (ITAM) or Immunoreceptor Tyrosine-based Inhibitory Motifs (ITIM) are located directly in the intracellular cytoplasmic domain (22). The FcγRIIc gene is expressed on NK cells only with 13Q allele due to the glutamine (Q)/stop (STP) polymorphism at codon 13 located in the first extracellular domain (23, 24).

FcγRIII family is encoded by 2 genes (*FCGR3A* and *FCGR3B*) for the receptors (FcγRIIIa and FcγRIIIb). Although these receptors are also low affinity binding, they can both bind efficiently to multimeric IgG and immune complexes. FcγRIIIa receptor can also bind with intermediate affinity and expressed on monocytes, tissue specific macrophages, dendritic cells, NK cells and gamma/delta T cells (21). The *FCGR3B* gene, encodes a glycosylphosphatidylinositol (GPI)-anchored receptor, which is highly expressed on neutrophils (25). These FcγR proteins bind to different IgG subclasses and regulate immunity by causing cell activation or inhibition depending on the receptors engaged (16). The mechanisms for Fc-mediated activities include antibody-dependent cellular cytotoxicity (ADCC), antibody-dependent cellular phagocytosis (ADCP), antibody-dependent cell-mediated virus inhibition (ADCVI), antibody-dependent complement deposition (ADCD), aggregation, and immune activation (26–28). They also induce cytokine production *via* their immune activating or inhibitory motifs (22). Thus FcγR polymorphisms may influence diverse effector functions such as cytotoxicity, phagocytosis, cytokine production, antigen presentation and degranulation and may contribute to the outcome of infections (29). For instance, in encapsulated bacterial infections, FcγRIIa-131H may be involved in efficient clearance of IgG2-coated particles since it has a higher binding affinity for IgG2 (30–32). Conversely, homozygous FcγRIIa-131R genotype has been associated with severe forms of encapsulated bacterial infection (30, 31, 33, 34).

The allelic differences that affect FcγR function are also important in host immune mechanisms against viral infections. FcγRIIa-131H/H homozygous infants were found to be more susceptible to perinatal HIV transmission (35) but in other studies no associations between FcγRIIa genotypes and HIV infection rates were noted (36), suggesting further investigations are needed. In HIV-1 patients, FcγRIIIa-V176F genotype is associated with the development of Kaposi’s sarcoma and cryptococcal disease (12, 37, 38). Other studies have implicated the FcγRIIa-R131 allele in Dengue (39–41), and other viral infections (34, 42, 43). FcγRIIb inhibits activation signals from activating FcγRs (18) and the FcγRIIb-232T allele elicits reduced inhibitory signaling and has been associated with inflammatory diseases (44), however, it has not been extensively studied in HIV (45). These studies show that different FcγR polymorphisms influence effector functions in diverse ways and subsequently impacts on infection outcomes differently (**Table 1**).

**Table 1:** Studies of FcγR polymorphisms and infection outcomes.

Population from	Type of study	Sample size	Receptor	Main outcome	Reference
Kenya	In vitro	250 female sex workers	FcγRIIa (rs1801274) and FcγRIIIa (rs 396991)	No association with HIV-1 disease progression, viral set point or CD4 decline. Examined with individuals with FcγRIIa 131-H/R or H/H and FcγRIIIa 176-F/V, F/F or V/V genotypes.	(46)
USA, African Americans	Genotyping	172 HIV-1 progressors and natural viral controllers	FcγRIIa (rs1801274) and FcγRIIIa and (rs 396991)	FcγRIIIa-V176 but not FcγRIIa-H131 was significantly associated with HIV-1 disease progression.	(47)
Kenyan	Genotyping	379 mother-infant pairs	FcγRIIa (rs1801274) and FcγRIIIa (rs396991)	Infant FcγRIIa and FcγRIIIa were not associated with risk of HIV-1 infection or disease progression. Risk of transmission increased with maternal FcγRIIIa.	(48)
India	Genotyping and *in vitro*	63 HIV-1 infected individuals and 76 HIV-1 controls	FcγRIIIa (rs396991 and rs396716)	FcγRIIIa-V176F (rs396991) and Y158H (rs396716) genotypes significantly associated with higher HIV-1 specific ADCC response.	(49)
USA	Genotyping	559 HIV-1 infected males	FcγRIIa (rs1801274) and FcγRIIIa (rs396991)	Association with risk of HIV-1 infection progression and faster rate of CD4 decline for FcγRIIa-131RR. FcγRIIIa-V176F alleles were associated with risk of Kaposi’s sarcoma.	(12)
Rwanda and Zambia	Genotyping and *in vitro*	836 HIV-1 infected Heterosexual sero-discordant couples	FcγRIIa and FcγRIIIa	No clear FcγRIIa-H131R and FcγRIIIa-V176F association with time to HIV-1 acquisition, viral load in early infection, or CD4+ T-cell decline over time after infection.	(50)
Paris	In vitro	12 HIV-1 infected individuals	FcγRIIa (CD32a)	A marker for HIV-1 reservoir.	(51)
Thailand	In vitro	125 HIV-1 infected individuals	FcγRIIc (rs114945036)	Associated with protection from HIV-1 infection in RV144 vaccine recipients in individuals carrying the *FCGR2C* 126C>T genotypes.	(13)
Spain	In vitro	23 HIV-1 infected males	FcγRIIa (CD32a)	A marker for T cell activation, but not for HIV-1 reservoir.	(52)
USA	In vitro	58 HIV-1 infected males and females	FcγRIIa and FcγRIIIa	All genotypes were associated with enhanced FcγR signaling in HIV-1 viremic controllers.	(53)
South Africa	In vitro	193 HIV-1 infected and control subjects	*FcγRIIc (rs138747765, rs78603008, rs373013207 rs201984478) and FcγRIIIb (rs34322334, rs61803026, rs34085961)	*FCGR2C*-TATA and *FCGR3B*-AGA haplotypes increased the risk of HIV-1 acquisition in some HVTN 505 vaccinees but not in others.	(54)
Kenya	In vitro	448 HIV-1 seropositive women and their infants	FcγRIIa (rs1801274)	Infant FcγRIIa-H131H genotype was associated with risk of perinatal HIV-1 transmission.	(35)
Kenya	In vitro	903 pregnant women	FcγRIIa (rs1801274)	FcγRIIa-131H/H genotype associated with high risk of placental malaria in HIV-1 positive women compared to HIV negative women.	(55)
Europe	Genetic association studies	7,247 population samples	FcγRIIa (rs1801274) and FcγRIIIa (rs396991)	No association of these polymorphisms in HIV-1 acquisition	(56)
USA	In Vitro/In vivo	1725 male subjects	FcγRIIa (rs1801274) and FcγRIIIa (rs396991)	Homozygous FcγRIIIa V176 allele individuals were more likely to acquire HIV -1 among vaccinees. No association of FcγRIIa genotype and HIV-1 infection rate	(36)
South India	In vitro	120 Periodontitis subjects and controls	FcγRIIIa (rs396991)	FcγRIIIa-V176V genotype may be a risk factor for chronic periodontitis	(57)
USA	In vitro	250 male subjects	FcγRIIIa (rs396991)	FcγRIIIa-V176F genotype significantly associated with the risk of developing Kaposi’s sarcoma during HIV-1 infection.	(37)
USA	In vitro	164 HIV infected cases and controls	FcγRIIIa (rs396991)	An association between the FcγRIIIa-176V allele and risk for HIV-1 associated cryptococcal disease.	(38)

*FCGR2C-TATA haplotype (minor alleles of 4 SNPs; FCGR2C-exon06-441-C/T (rs138747765); FCGR2C-intron06-590-G/A (rs78603008), FCGR2C-intron15-403-C/T (rs373013207) and FCGR2C-intron15-433-G/A (rs201984478).

FCGR3B-AGA haplotype (minor alleles of 3 SNPs; FCGR3B-5’utr44-T/A (rs34322334), FCGR3B-5’utr99-C/G (rs61803026), and FCGR3B-5’utr222-G/A (rs34085961).

## FcγR Polymorphisms and the Risk of HIV-1 Infection With and Without Vaccines

Studies have not provided conclusive data on the association between FcγR polymorphisms and HIV-1 infection (56). For instance, contrasting results were reported when FcγRIIa and FcγRIIIa were examined with the risk of perinatal HIV-1 infection among infants in Kenya (35, 48). Whereas Brouwer et al., found that infant FcγRIIa-H131H (rs1801274) genotype was associated with susceptibility to perinatal HIV-1 transmission, Milligan and colleagues observed no such association with both FcγRIIa and FcγRIIIa. Rather, they observed that maternal FcγRIIIa-V176F genotypes may lead to higher risk of mother to child transmission compared to the homozygous (V/V or F/F) genotype carriers. The differences in these results could be attributed to differences in the cohort design as well as statistical rigor (48). More work is needed to define the role of these genotypes in mother to child HIV-1 transmission. Such a study may involve using harmonized protocols in a multi-center recruitment of a reasonably large number of HIV-1 infected pregnant women, determining their FcγR genotypes and monitoring their viral load throughout the pregnancy till birth. The HIV-1 infection status and FcγR genotypes of the child could then be determined to assess the genotype association with mother to child HIV-1 transmission.

A study that used samples from the European Multicenter AIDS Cohort Study (MACS), found an association between FcγRIIa-131R/R genotype and a faster rate of CD4+ T cell decline and disease progression compared to individuals with the R131H or H131H genotypes (12). This may be due to the weaker binding of the 131R/R receptor to IgG2 and IgG3 immune complexes. In-vitro experiments have shown that monocytes bearing this receptor do not efficiently internalize HIV-1 complexes, compared to 131H/H receptors (12, 58). Furthermore, the expression of FcγRIIa on immune cells leads to the activation and production of proinflammatory cytokines, an indication that FcγRIIa-mediated T-cell activation would be more efficient in individuals carrying the FcγRIIa-H/H genotype (59, 60). This suggests that FcγRIIa polymorphism may also indirectly influence CD4+ T cell function, and subsequent disease progression through its effect on immune complex internalization by monocytes and dendritic cells leading to their activation (59, 60).

Surprisingly, the 131R/R genotype was found to be associated with decreased AIDS-induced pneumonias compared to the 131H/H genotype in the MACS cohort. Given that FcγRIIa also binds to C-reactive proteins (61), it is possible that during bacterial infections, carriers of the 131R/R genotype may have higher levels of CRP to opsonize IgG2-coated microbes and activate the complement receptors to clear infection (62).

A recent study showed that HIV-1 patients with homozygous 176V for the FcγRIIIa-V176F (rs396991) polymorphisms and/or Y158H (rs396716) genotypes have higher HIV-1 specific ADCC response (49). It was hypothesized that the V176F polymorphism improves the binding capacity between the Fc receptor and anti-HIV-1 antibody, indicating that the FcγRIIIa receptor expressed on NK cells induced strong ADCC response for viral clearance (63, 64). When Geraghty et al. examined the role of FcγRIIa*-*(rs1801274) and FcγRIIIa*-*(rs396991) polymorphisms in 6300 HIV+ adults with European ancestry from a previous GWAS (65), they did not find any association between these polymorphisms and HIV acquisition (56). Furthermore, in the largest study of its kind in Africa, Connolly and colleagues also found no association between FcγRIIa*-*H131R (rs1801274) and FcγRIIIa*-*V176F (rs396991) variants and HIV infection or CD4+ T-cell decline in two cohorts in Rwanda and Zambia (50).

Accumulating evidence suggest that FcγR polymorphisms may play a key role in HIV-1 acquisition when vaccines are involved (13, 36, 54, 66–68). In the recombinant HIV-1 glycoprotein (gp120) Vax004 vaccine trial in the USA, those homozygous for the FcγRIIIa V176 allele in the lowest behavioral risk group were more likely to acquire HIV compared to individuals carrying the FcγRIIIa 176F or V176F genotypes in the same behavioral risk group (36). However, in that same study, no FcγRIIa*-*H131R (rs1801274) genotype was associated with increased risk of HIV among vaccine and placebo recipients. Furthermore, in the RV144 trial in Thailand, a SNP in *FCGR2C* (126C>T, rs114945036) was associated with vaccine efficacy. The study found an 11 to 15% efficacy in CC subjects compared to 64 to 91% in CT/TT subjects with the HIV subtype CRF01_AE 169K HIV-1 (13). On the contrary, in the HVTN 505 vaccine trial, the effect modification of this SNP with respect to vaccine efficacy was not significant. However, other four SNPs; (*FCGR2C*-exon06-441-C/T, rs138747765, *FCGR2C*-intron06-590-G/A, rs78603008, *FCGR2C*-intron15-403-C/T, rs373013207 and *FCGR2C*-intron15-433-G/A, rs201984478), were associated with increased risk of acquiring HIV in those vaccinated compared to the placebo group (54). These studies provide evidence that FcγR polymorphisms impact HIV-1 infection outcomes differently in vaccine recipients. The affinity of different IgG isotypes and the expression pattern of these FcγR on effector cells could account for these differences. For instance, when neutralizing antibodies induced during HIV-1 vaccination (69, 70) are ineffective against the virus, the antibody-virus complexes formed could lead to antibody dependent enhancement, resulting in virus spread among cells expressing these receptors (71–73).

It is also possible that vaccine types and their modes of delivery could trigger different FcγR responses. For instance, the RV144 vaccine consists of recombinant canarypox vector containing HIV antigens and recombinant gp120 administered in a prime-boosted regimen, while the HVTN 505 vaccine was made of a recombinant adenovirus serotype 5 vector boost (DNA/rAd5). The HVTN 505 vaccination increased the risk of HIV-1 acquisition. In addition, there were additional FcγR SNPs in HVTN 505 (*FCGR3B* SNPs) that modified vaccine effect in relation to HIV-1 acquisition compared to the RV144 vaccine recipients who had no such SNP associations (54). Furthermore, some minor SNPs identified on *FCGR2C* intron 6 were found to be in high linkage disequilibrium with others. This SNP may have selectively modulated the expression of FcγR effector functions differently in the two vaccines mentioned above (54). The *FCGR2C* polymorphisms, which was found in Thai RV144 vaccinees (13), was absent in Africans. This has implications for determining vaccine responses in other populations, if these *FCGR2C* polymorphisms are used as a proxy for FcγRIIc expression (45). Hence, it is possible that ethnic group, vaccine types and methods for delivering the vaccines may all trigger differences in FcγR dependent responses.

It has been proposed that during vaccination, FcγR polymorphism influence HIV-1 acquisition through their effect on the innate immune response. During HIV-1 infection, plasmacytoid dendritic cells produce high levels of interferon, which restricts the replication of virus (74). However, when viral particles are opsonized, there is suppression of type I and III interferons that are produced *via* FcγR-mediated mechanisms. This results in the lowering of the interferon levels required to block infection (54, 75).

FcγR polymorphisms also determine the affinity to the Fc region of IgG and alter its functionality after vaccination. For instance, in the HVTN 505 vaccine trial, differences in FcγRIIa binding and gp140-specific ADCP activity among the vaccine and control groups occurred in only one genotype of the FcγRIIa and FcγRIIb SNPs (54). This suggests that FcγR polymorphisms influenced the variation observed in the Fc region of the IgG induced after HVTN 505 vaccination (54). Also, studies have shown variation in Fc glycosylation of HIV-specific antibodies in HIV-1 infected patients and vaccine recipients, an indication that it regulates antibody and FcγR interaction (76, 77). Although these findings suggest possible mechanisms associated with increased risk of HIV-1infection in HVTN-505 vaccine among individuals carrying certain FcγRIIc genotypes, the same effect could not be established among recipients of the RV144 vaccine. These studies underscore the need to consider FcγR polymorphisms in HIV-1 vaccine trials, since they regulate vaccine-induced immunity, which impacts on HIV infection outcomes (36, 54).

## FcγR Polymorphisms, HIV Disease Progression and ART Outcomes

Several studies have evaluated FcγR polymorphism and infection progression in European populations, however, studies on FcγR polymorphisms and ART outcomes are lacking. FcγR polymorphisms have been shown to affect the viral reservoir size in acute HIV-1 patients who were put on early ART (78, 79).

In a meta-analysis of several genome-wide association studies comprising 7,266 patients in the International Collaboration for the Genomics of HIV (ICGH), no association between FcγRIIa (rs1801274) and FcγRIIIa (rs396991) polymorphisms and viral set point was found [reviewed in (56)]. Similarly, in a sub-analysis of 467 long-term non-progressors and 517 rapid progressors, there was no association between FcγRIIa polymorphisms and HIV-1 disease progression. Finally, in the same meta-analysis, FcγRIIIa polymorphisms were not associated with HIV-1 disease progression in the Swiss HIV Cohort Study (SHCS) (56). Although another study found homozygous FcγRIIIa-176V/V to be highly prevalent in HIV progressors on ART compared to untreated natural viral suppressors, they could not conclude on this polymorphism’s association with viral set point (47). This is probably because the use of ART for HIV progressors in this study influenced their viral load, hence analysis with this polymorphism with respect to viral load could not be substantiated. Similarly, a study conducted in 253 Kenyan women to evaluate the impact of FcγRIIa-131H/R genotypes and FcγRIIIa-176F/V polymorphisms on HIV-1 disease progression could not find an association between these polymorphisms with viral load set point, decrease in CD4 count or increase in viral load (46).

The studies above agree that FcγRIIa and FcγRIIIa genotypes have no effect on viral load set point, but the results on disease progression differ. The divergent results on disease progression could be due to sample size, different study populations (i.e., men versus women), clinical definitions and the rigorousness of statistical methods employed. Additionally, the differences in these genotypic profiles among the different study populations and association with infection progression may be due to other factors such as viral type, host immunity and genetics. Future studies investigating the role of FcγRs in HIV-1 disease progression should comprehensively address potential population substructure, and longitudinally, pre-existing neutralizing antibodies and genetic variability in the virus to ascertain how these variables influence the outcome. Though haplotype analyses within and between the FcγR genes may offer crucial information on why these differences exist, such studies have been few (11, 80). Differences within the FCGR locus for different populations have been established (81–85). For instance, FcγRIIIa-176V was found to be underrepresented in Kenyan population (23.7%) compared to Europeans and Dutch Caucasians, whiles FcγRIIa-131H and FcγRIIc were highly prevalent in Asians compared with Caucasians (45). In addition, the distribution of FcγRIIIb-HNA1a/HNA1b allotypes were different among different populations, however, the FcγRIIIb-HNA1a and FcγRIIb- 232T variants were highly prevalent in black South Africans compared with Caucasians (45). Different effector functions have been observed in these populations with respect to enhanced cell activation and neutrophil-mediated phagocytosis as a result of these polymorphisms (45). Furthermore, gene copy number variations (CNVs) in FcγRIIc, FcγRIIIa and FcγRIIIb were shown to play a key role in association with HIV-1 infection and ART outcomes (45, 80).

Most of the studies on FcγR polymorphisms were conducted in Europe, USA, Asia and a few in South Africa. Data on the impact of FcγR polymorphisms and CNV on HIV-1 infection are limited in most African populations (12, 13, 45). One study examining the effect of FcγRIIc, FcγRIIIa and FcγRIIIb CNV in Ethiopian and Tanzanian cohorts found no effect on immune reconstitution post ART (80). Thus, supporting the limited role of poorly neutralizing or non-neutralizing antibodies in HIV-1 control (67, 86). This was contrary to the hypotheses that different FcγRs play a key role in mediating a balance between activating and inhibitory functions, IgG binding affinity to receptors and antibody mediated responses in HIV-1 infection progression. These findings in the Tanzanian and Ethiopian cohorts could also be due to epistatic interactions between these FcγR variants and IgG affinity, that will mediate HIV pathogenesis as evidenced in KIR/HLA variants (87).

Importantly, data is lacking on FcγR influence on HIV-1 disease progression and ART responses in African populations, though the continent bears the brunt of the epidemic. Therefore, large studies conducted in populations with African ancestry are needed. This will provide increased power to detect population specific genetic variations in association with disease progression, when combined with data from European populations (11, 88).

## FcγR Polymorphisms and Viral Reservoir

Though ART has been used successfully in the management of HIV-1 infection, replication-competent viruses persist as latent reservoirs after long term ART usage (89). Even HIV-1 elite controllers and long term non-progressors, harbor viral reservoirs (9, 10). The extent of viral clearance in HIV-1 infection, will be dependent on both an efficient HIV-1 specific immune response and a very low reservoir size (90). To achieve an HIV cure or remission, the viral reservoir must be eliminated or reduced to a minimum, since the size of the viral reservoir has been shown to be a marker for disease progression and clinical outcomes (91, 92). Therefore, reduction of the reservoir can be used as a criterion for ART interruption in HIV-1 cure studies (92). The impact of FcγRs on ART outcomes needs further evaluation, since it has been hypothesized that some polymorphisms affect the viral reservoir size in patients with acute HIV-1 infection initiated on early ART (78, 79). However, such studies analyzing FcγRs polymorphisms and HIV reservoir size are currently lacking.

There is now increased focus on identifying markers for these latent reservoirs to help in the HIV-1 cure efforts. Receptors expressed on infected cells such as CD30 and CD32 (FcγRIIa) and some immune checkpoint inhibiting molecules on the surfaces of infected cells have been identified as potential markers for latently infected cells (51, 52, 93). Recently, CD32a (FcγRIIa) was identified as a marker of latently infected CD4 T cells. In this study CD4+ T cells expressing CD32a+ molecules were observed to be highly enriched in HIV-1 DNA and contained replication competent proviruses compared to the CD32a- CD4+ T cells (51). However, these findings were not replicated in other studies. Some investigators reported lower enrichment for HIV-1 DNA in cells expressing CD32 in certain individuals (52, 93). Others could not verify that CD32a is expressed on latent reservoirs, nor enriched in CD4+ T cells carrying viral DNA (94–96). A study that used 10 chronic HIV-1 patients on ART also observed no enrichment for HIV-1 DNA in CD32+ CD4 T cells (95). Some investigators proposed that the CD32+ cells may have been derived from adherent non-T cells and other cell conjugates (T-B cell conjugates) expressing this marker (95). Another study proposed that CD32 (FcγRII) is not a specific biomarker for most CD4 T cell populations because they found a greater number of HIV-1 latent reservoirs occurred in CD3+CD4+ CD32- T cells using a quantitative viral outgrowth assay (qVOA) (97).

Some studies have suggested that the inconsistencies in these findings could be attributed to cell sorting techniques that was not able to isolate pure CD4 T cells that express only CD32+ marker, but rather T-B cell doublets (96, 98). In addition, it has been shown that there is a higher proportion of antigen presenting cells (APC) expressing CD32+ cells than what is expressed on CD4+ T cells. Therefore, high number of residual non-T cells found in sorted CD32+ cell population, can bias these findings (93, 95, 99, 100). The cell sorting challenges seem to have been overcome by Darcis and colleagues using an improved isolation and purification technique. They performed two rounds of CD4+ T cell negative selection by magnetic cell sorting before CD32 isolation. Using this method, they observed an increased HIV-1 DNA enrichment in the CD32+ CD4+ T cells (100) as seen in the original study by Descours et al.

Further studies that replicate the sequential cell sorting techniques used by Darcis and colleagues are needed (99) to firmly establish FcγRIIa as bona fide marker of latently infected CD4+ cells. After that, several questions still remain: Is FcγRIIa just a marker of these latently infected cells or does it have functional consequences on the size or reactivation capacity of the reservoir? Do polymorphisms and CNVs in FcγRIIa and other Fc receptors determine the size of the reservoir in individuals treated with ART? Does it matter if the persons are treated early during HIV infection? What about the tissue reservoirs, do they also express FcγRIIa and if so, do polymorphisms and CNV matter in that context? Investigators have started to address some of these important questions. Some have hypothesized that the differences in reservoir size in people who started early on ART may be due to polymorphisms in FcγRIIa (78, 101), since there has been previous association of this gene with HIV-1 disease pathogenesis. However, this hypothesis needs to be tested. Notably, these reservoirs are diverse in nature, occurring in various infected cells and tissues, and thus may enhance the long-term persistence of replication competent viruses (99, 102, 103). Also, since there is some evidence that CD32 (FcγRIIa) marks transcriptionally active HIV-1 infected cells, they could be used to identify persistent HIV-1 infected CD4 T cells that may contribute to viral persistence during antiretroviral treatments (104, 105). The co-expression of CD32a (FcγRIIa) with other markers such as PD-1 in lymph node CD4+ T cells (105) as well as CD32a occurring with CD30 in CD4+ T cell tissues (104), shows these reservoirs are heterogenous in nature.

In a previous study of HIV controllers and non-controllers, epistatic interactions between of genes (GM on chromosome 14) encoding variability in the Fc portion of IgG and FcγR genes (on chromosome 1) were reported to influence the control of HIV-1 viral replication in Caucasian Americans (106). Homozygous FcγRIIa-H individuals who were also GM21 non carriers (homozygous GM5) were more likely to be HIV controllers than GM21 carriers. A similar interaction between GM determinants and FcγRIIIa alleles on HIV control albeit mush weaker compared to that with FcγRIIa. Interestingly, these epistatic interactions between FcγR and GM genes were not observed in the African-American population in the same study highlighting population specific effects of these genes in HIV control (106). In a more recent study, the FcγRIIa AA genotype (rs10800309) was found to increase FcγRIIa expression on myeloid cells and was associated with HIV-1 control independent of HLA-B57 and HLA-B27 (107), which are well established markers involved in controlling viral load and long-term non-progression of HIV infection (108, 109). Taken together, these studies underscore the importance of studies assessing the role of FcγR polymorphisms in HIV to be designed to adequately address inter-ethnic differences in these genes and the potential impact of genetic variability affecting not only the receptor-ligand interphase but also receptor expression levels.

## FcγRs, Broadly Neutralizing Antibodies and HIV-1 Remission Attempts

Investigators are exploring different approaches to achieve a remission or cure for HIV (**Table 2**). First, there are strategies that seek to boost the host immune system using vaccines that produce broadly neutralizing antibodies (bNAbs) to suppress the virus, or chimeric antigen receptors to engage and destroy the latently infected cells. Second, genetic methods like the CRISPR/Cas9 gene editing technology to excise or mutate the integrated virus or render patients resistant to HIV by taking their CD4+ T cells, mutating essential receptors such as CCR5 and reinfusing them back into the patient (126–128). Third, and probably the most studied of the HIV cure methods is the shock and kill approach which seeks to use compounds to reactivate the latent virus with the hope that infected CD+ T cells will die by viral cytopathic effects or immune clearance (129–132). The use of bNAbs, chimeric antigen receptors, and the ‘kill’ part of the shock and kill approach may all involve the engagement of Fc gamma receptors. FcγR binding seems to be a key requirement for bNAbs to efficiently protect from infection, viral suppression, and clearance. Studies conducted in animal models observed a decrease in broadly neutralizing antibody activity from simian-human immunodeficiency virus (SHIV-1) challenge when the FcγR and complement activities were disabled (118). Similarly, bNAbs enhanced binding capacity for activating FcγRs FcγRIIa and FcγRIIIa, which were demonstrated to be protective in humanized murine models (133).

**Table 2:** Vaccine and bNAb studies.

Research Type	Type of study	Performed in	Mechanism of Action	Main Outcome	Reference(s)
Phase 1 HIV-1 Clinical Trial	In vivo/In vitro	USA volunteers	Targets CD4-binding site of the HIV-1 envelope glycoprotein.	Fc modified -VRC01LS (bnMAbs) vaccine was well tolerated, with 4-fold increase in half-life, compared to wild type.	(66)
RV144 ALVAC-HIV	In vivo/In vitro	USA volunteers	RV144 HIV-1 vaccine protection partially due to ADCC-mediating antibodies.	Antibody binding to envelope V1V2 was associated with HIV-1 infection risk.	(67, 110–116)
Phase III RV144 ALVAC/AIDSVAX clinical trial	In vivo/In vitro	Thai volunteers	Vaccine-induced FcR-mediated antibody function was associated with reduced risk of HIV-1 infection	*FCGR2C* tag SNP (rs114945036) was associated with VE against HIV-1 subtype CRF01_AE. Individuals with CC SNPs had 15% vaccine efficacy compared 91% of those carrying CT or TT.	(13)
bNAbs	In vitro	Cell lines	Impair clustering and fusion of infected and target cells, blocked the transfer of virions to uninfected T cells	VRC01, NIH45-46 bNAbs prevented HIV-1 cell-to-cell transmission	(70)
bNAbs	In vivo/In vitro	humanized mouse models	Fc domain-engineered bNAb variants 3BNC117 (GASDALIE) and mouse IgG1 variant (D265A)	Fc domain engineering of anti-HIV-1 bNAbs enhanced interaction with activating FCGRs in an in-vivo model of HIV-1 entry.	(117)
bNAbs	In vivo/In vitro	Rhesus macaque model	KA (K322A) variant blocks complement activity but active in FCGR binding. The L234A, L235A (LALA) variant is defective of both complement and FCGR binding	Decreased protection of bNAbs against SHIV challenge when Fc receptor and complement-binding activities were engineered out of the antibody.	(118, 119)
bNAbs	In vivo/In vitro	Macaque model	WT bNAbs PGT121 and a LALA mutant of PGT121 (impaired Fc-dependent function) was experimented for ability to protect pigtail macaques	Potent neutralizing capacity of PGT121 renders the Fc-dependent functions of the Ab are less effective	(120)
mAbs	In vitro	Swiss volunteers	use of neutralizing monoclonal antibodies, gp41 and mAb VRC01 in blocking HIV-1 infectivity	CD4bs mAbs blocked free virus transmission but allowed HIV-1 to spread among cell-cell contacts.	(121)
Antibodies	In vitro	Australian volunteers	ADCC to activate NK cells, either from HIV-1 positive or healthy donors.	NK cells from the HIV+ subjects induced ADCC responses to either gp140 Env protein or HIV-1 peptide pools	(122)
Vaccine trial	In Vitro/In Vivo	Americans	Infected immune complexes easily bind, enter, and infect susceptible FcγRIIIa-176V/V genotypes to establish infection	Homozygous FcγRIIIa V176 allele individuals were more likely to acquire HIV-1 among gp120 vaccinees.	(36)
Vectored Immunoprophylaxis	in Vitro	Humanized mouse models	Use of vectored monoprophylaxis (VIP), a specialized adeno-associated virus vector that produces full-length antibody and induces lifelong expression of these monoclonal antibodies.	Humanized mice were fully protected from HIV-1, when challenged intravenously with very high doses of replication-competent virus	(123)
HIV-1 Gag-Pol vaccine	In Vitro	Australia	Use of recombinant fowl pox virus inserted with HIV Gag-Pol genes and interferon gamma to control HIV-1 after ART is ceased.	There was lower replication of HIV-1 in patients with IgG2 anti-p24 and carriers of FcγRIIa-131 R/H polymorphisms	(68, 124, 125)

bNAb, broadly neutralizing antibodies; mAb, monoclonal antibodies.

In contrast, though both the WT PGT121 and LALA PGT121 bNAbs administered to macaques, conferred protection against cell associated SHIV SF162P3 challenge, when tested *in vitro*, the Fc-dependent function was greatly reduced. Although, these two bNAbs have been shown to be associated with reduced plasma viremia both in macaques and humanized mice models (133, 134), indication that the protection conferred by PGT121, may not be dependent on Fc-mediated NK cells responses (120, 135, 136). It has been suggested that the mechanism by which these bNAbs neutralize both cell-free virus and cell-associated virus and confer protection could be through the fragment antigen-binding (Fab) region, as well as epitope specificity of these antibodies (120). Additional studies on other bNAbs and other modified bNAbs in different animal models are needed to validate these results (120). These findings have emphasized the differences in the formulation of bNAbs, and how it affects their function in HIV control. Delineating what influences bNAb function either more through an Fc-mediated mechanism or otherwise would be very important in HIV cure research aimed at using bNAbs as immune-therapeutic tool in effective control and management of HIV.

During HIV-1 infections, broadly neutralizing antibodies (bNAbs) coordinate with Fc receptors to activate effector cells to clear the virus through mechanisms such as ADCC (21, 67, 122). In addition, there is evidence that neutralizing antibodies block cell-free virus through a mechanism that prevents cell-cell transmission. For instance, certain classes of CD4 binding site inhibitors (CD4b) such as mAb VRC01 only block free virus but has low activity during cell to cell transmission (121). Other mAbs such as membrane-proximal external region (MPER), CD4bs and anti-coreceptor agents are involved in blocking free virus transmission (137, 138). Subsequent studies have shown that mAb VRC01 may engage FcγRs and prevent cell to cell HIV-1 transmission and also reduce the half-lives of infected cells and free virions (70, 121). While a potent HIV-1 vaccine must block cell free virus and cell-cell virus transmission (121), using two or more bNAbs that bind different epitopes in combination could enhance efficacy and offer broader protection against HIV-1 infections. This was demonstrated when the combination of CD4bs and VRC07 resulted in the neutralization of about 98% viruses in one study (139). This notwithstanding, the role of antibodies in cell-cell HIV inhibition is divergent, since these findings were based on experiments conducted with several viral strains and antibodies, different donor and target cell types, as well as various HIV-1 transmission models (140). Hence some studies have reported similar HIV-1 inhibition for cell-free virus compared to cell-to-cell transmission (141–143), while others observed a decreased antibody effect in cell-to-cell transmission compared to cell free virus inhibition (70, 121, 144). These controversies appear to have been resolved in comparative studies that obtained similar results when experimental designs were normalized with respect to target cells used, and antibody inhibition activities (141–143, 145). Thus, normalization of these factors must be done in terms of the type and quantity of virus and cell types using standardized assays to establish the role of these antibodies in cell free or cell-to-cell HIV transmission (140).

FcγR differences could also affect the efficacy of bNAbs as HIV-1 prevention agents. In animal studies, bNAbs confer protective immunity against HIV-1 or SHIV-1 challenge in humanized mouse models (Hessell et al., 2007, Balazs et al., 2012, Lu et al., 2016). Recently, Simone and colleagues observed that among a cohort of 23 individuals, 13 developed broadly neutralizing antibodies to HIV-1, which was significantly associated with antibody binding to FcγRIIa. These antibodies were associated with higher Fc polyfunctionality early in the course of infection (146). Furthermore, FcγRIIIa-V176F (rs396991) and FcγRIIIa-Y158H (rs396716) polymorphism are associated with enhanced ADCC in HIV-1 patients (49), giving the indication that the expression of FcγRIIIa receptor on NK cells led to the induction of strong ADCC response for viral clearance (63, 64). These studies suggest that bNAbs have potential as therapeutic or prophylactic treatment in humans. It is becoming clearer that if bNAbs are to become successful in eliminating the reservoir, FcγR-mediated actions will play a pivotal role. It is therefore imperative to determine at this early stage if polymorphisms and CNVs affect how bNAbs can suppress or eliminate HIV-1 in different populations. This is especially crucial since inter-ethnic variations have been reported for polymorphisms in FcγRIIa that influences its affinity for IgG binding (45, 147, 148).

## Perspectives and Conclusion

Although several studies have evaluated the impact of FcγR polymorphisms on HIV progression, they do not appear to play a major role in viral load set point or natural control of the virus. However, when it comes to HIV vaccine responses and remission attempts, FcγR polymorphisms and/or CNVs may play a variety of roles. First, if FcγRIIa (CD32) is confirmed as a true marker of the latent reservoir, it will raise many crucial questions as enumerated above. One such critical question is whether FcγR polymorphisms determine the size of the viral reservoir. Second, the use of bNAbs either for reservoir elimination or long-term suppression may depend on engagement with FcγRs. It is important to determine if expression of different variants of FcγRs determine why these antibodies are successful in some persons but not others. Also, it will be significant to determine if FcγRs play a role in the development of resistance or immune tolerance to these antibodies. Third, FcγRs may need to be engaged in cure methods such as the ‘shock and kill’ approach, which seeks to reactivate the reservoir for clearance by the immune system. The ‘kill’ portion of this approach may involve interventions such as bNAbs or chimeric antigen receptors. The role of FcγRs in mediating successful killing of T cells that have been reactivated also need to be investigated especially after ART initiation as well as the synergistic effect between FcγRs and ART outcomes need to be evaluated. Finally, the responses to most HIV-1 vaccines are likely to be dependent on FcγRs as the few unsuccessful vaccine trials have shown. Therefore, investigations into FcγR variations in different populations offer an important area of inquiry in the HIV cure research era.

## Author Contributions

HL, BA, and GK designed the review and the concepts. EB assisted in the literature search and manuscript writing. All authors wrote and reviewed the manuscript. All authors contributed to the article and approved the submitted version.

## Funding

This project is part of the EDCTP2 program supported by the European Union with grant numbers TMA2017SF-1955 senior fellowship to GK and TMA2018PF-2535 preparatory fellowship to HL. The funder had no role in the design, analysis, or publication of the study.

## Conflict of Interest

The authors declare that the research was conducted in the absence of any commercial or financial relationships that could be construed as a potential conflict of interest.

## Abbreviations

ART, Anti-Retroviral Therapy; ADCC, Antibody-Dependent Cellular Cytotoxicity; ADCD, Antibody-Dependent Complement Deposition; ADCP, Antibody-Dependent Cellular Phagocytosis; ADCVI, Antibody-Dependent Cell-mediated Virus Inhibition; bNAbs, Broadly neutralizing antibody; CD4 T cells, Cluster of Differentiation 4 T cells; CNVs, Copy Number Variations; DC’s, Dendritic Cells; EC, Extracellular Domain; FcγR, Fc gamma receptors; FCGR, Fc gamma receptor gene; GPI, Glycosylphosphatidylinositol-anchored; HLA, Human Leukocyte Antigen; HIV, Human Immunodeficiency Virus; ITAM, Immunoreceptor Tyrosine-based Activation Motif; ITIM, Immunoreceptor Tyrosine-based Inhibitory Motif; IgG, Immunoglobulin; IC, Intracellular Domain; NK, Natural Killer Cells; SNPs, Single Nucleotide Polymorphisms; TM, Transmembrane Domain.
